# Supplementary material for: Deep Sequencing of Early T Stage Colorectal Cancers Reveals Disruption of Homologous Recombination Repair in Microsatellite Stable Tumours with High Mutational Burdens
Source: Cancers (Basel). 2022 Jun 14;14(12):2933. doi: 10.3390/cancers14122933 (PMC9221133; doi:10.3390/cancers14122933)

# Supplementary Information

**Deep sequencing of early T stage colorectal cancers reveals disruption of homologous recombination repair in microsatellite stable tumours with high mutational burdens**


Jun Li[1], Pascal Steffen[1], Benita C. Y. Tse[1], Mahsa S. Ahadi[2,3], Anthony J Gill[2,3], Alexander F. Engel[3,4], Mark P. Molloy[1*]

1 Bowel Cancer and Biomarker Laboratory, Kolling Institute, School of Medical Sciences, Faculty of Medicine and Health, The University of Sydney, NSW 2006

2 NSW Health Pathology, Department of Anatomical Pathology, Royal North Shore Hospital, NSW 2065

3 Sydney Medical School, Faculty of Medicine and Health, The University of Sydney, NSW 2006

4 Department of Colorectal Surgery, Royal North Shore Hospital, NSW 2065

*Correspondence: m.molloy@sydney.edu.au


------------------------------------------------------------------------------------------------------------------------

**Table S1.** Gene frequency differences amongst 69 Stage I and 12 Stage IIIA CRC specimens from TCGA COAD-READ projects.

**Table S2.** Gene variants identified in TMB-H MSS CRC samples.

**Figure S1.** Oncoplot showing the most frequently mutated genes from non-hypermutators in TCGA COAD-READ project compared with TMB-low CRC in this study.

**Figure S2**. Lollipop plot shows distributions of *APC* variants in hypermutated and non-hypermutated TCGA COAD-READ cohorts.

**Figure S3.** Oncoplot of HRR genes in the ten hypermutators of TCGA COAD-READ cohort.

**Supplementary Figure S1**. Oncoplot showing the most frequently mutated genes* from non-hypermutators in TCGA COAD-READ project compared with TMB-low CRC in this study.

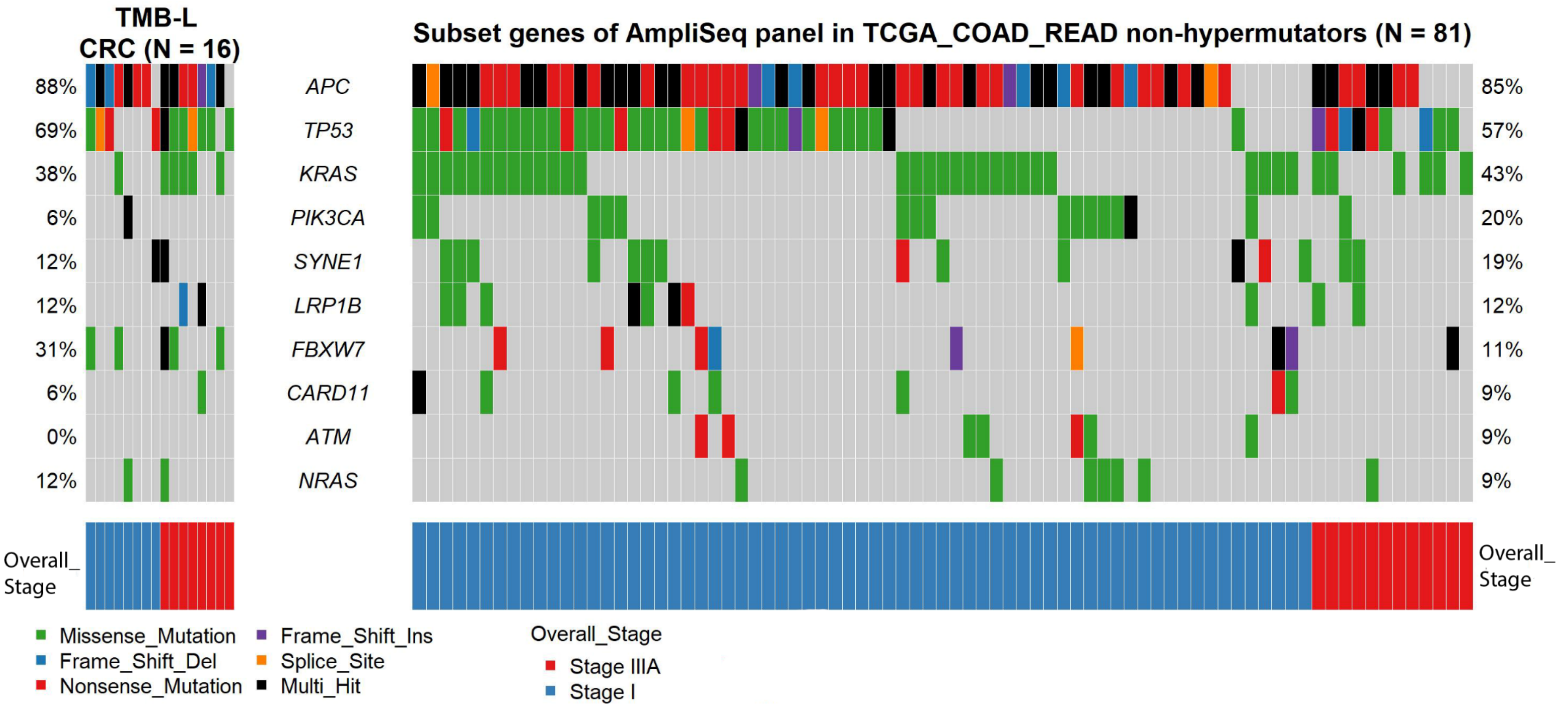


*Considering mutation files of TCGA-COAD-READ projects were generated via WXS, we hereby focused on the subset of 409 genes on the Ion AmpliSeq™ Comprehensive Cancer Panel in order to do meaningful comparison between our data and TCGA-COAD-READ data. These figures demonstrate observations between our study and TCGA are mostly consistent.

**Supplementary Figure S2**. Lollipop plot shows distributions of APC variants in hypermutated and non-hypermutated TCGA COAD READ cohorts

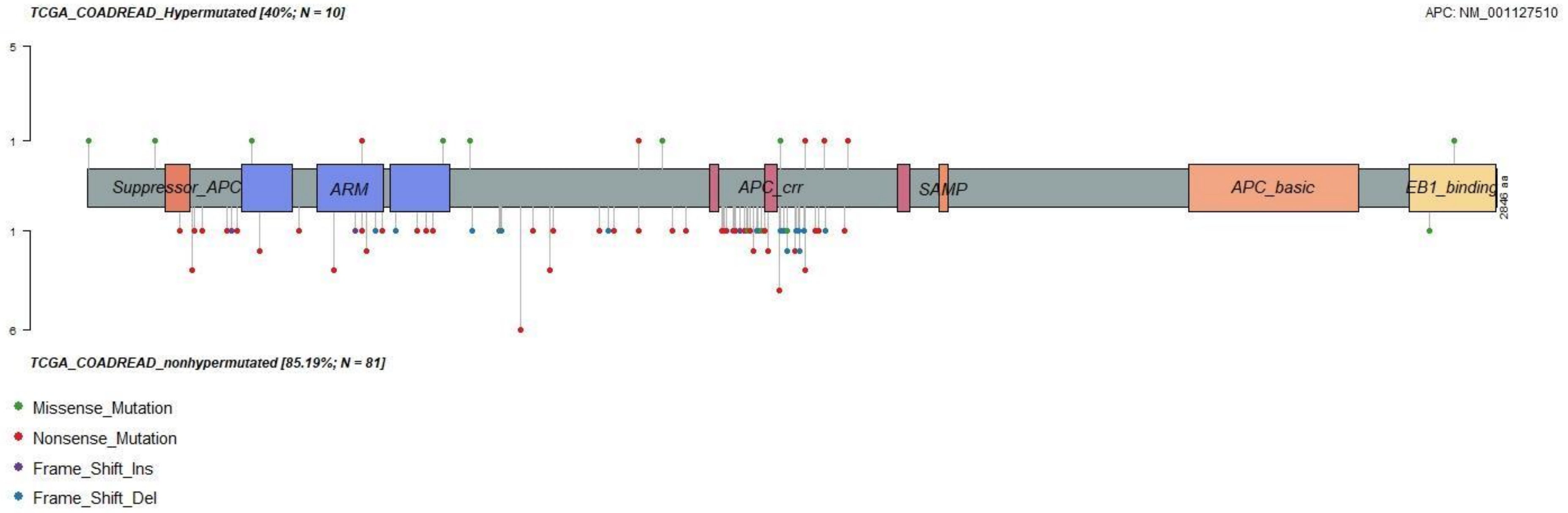

**Supplementary Figure S3**. Oncoplot of HRR genes in the ten hypermutators of TCGA COAD-READ cohort

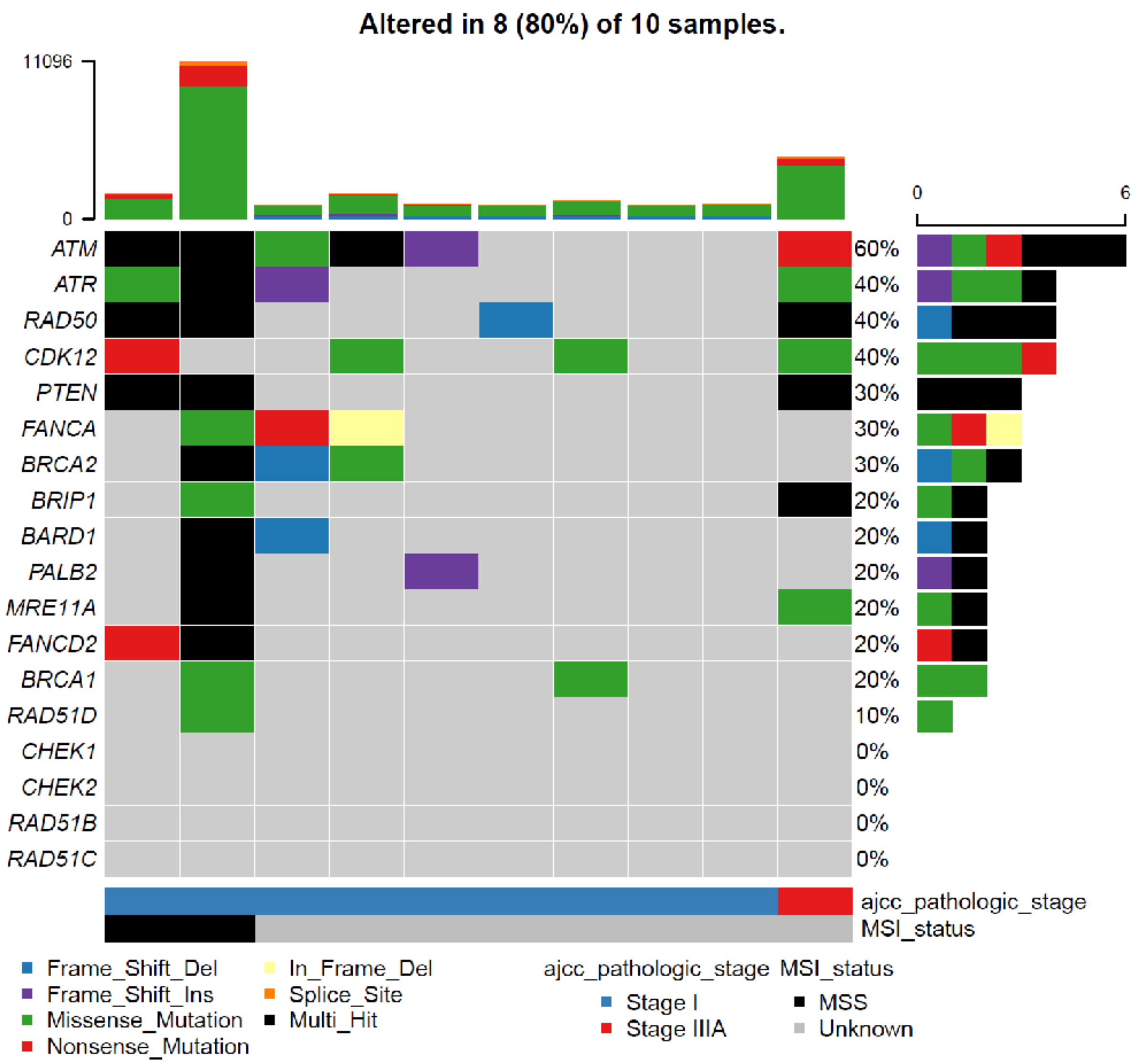

Supplement: Supplementary file 1 [file cancers-14-02933-s001.zip › Supplementary Information_Cancers_JunLi.pdf]
